# Age-related effects in magnitude and place-value processing

**DOI:** 10.1038/s41598-024-63298-z

**Published:** 2024-06-13

**Authors:** Christina Artemenko, Vaitsa Giannouli, Hans-Christoph Nuerk

**Affiliations:** 1 Department of Psychology, University of Tuebingen, Schleichstr. 4, 72076 Tuebingen, Germany; 2 LEAD Graduate School and Research Network, University of Tuebingen, Tuebingen, Germany; 3 Department of Psychology, University of Western Macedonia, Florina, Greece; 4 German Centre for Mental Health (DZPG), Tuebingen, Germany

**Keywords:** Aging, Older adults, Distance effect, Compatibility effect, Number magnitude comparison, Human behaviour, Psychology

## Abstract

While general cognitive skills decline during aging, numerical skills seem to be mainly preserved. Such skills are essential for an independent life up to old age, e.g., when dealing with money or time. Operating with numbers usually requires number magnitude and place-value processing. The question is whether these processes are negatively affected by aging due to the general cognitive decline or positively affected due to lifelong experience with numbers. Therefore, we investigated age-related changes in the distance and compatibility effects in single-digit, two-digit, and four-digit number comparison. On the one hand, older adults took longer for number processing and showed a smaller distance effect, indicating altered number magnitude representations. On the other hand, older adults were better in place-value processing as indicated by a smaller compatibility effect than in younger adults. We conclude that aging differentially affects basic numerical skills.

## Introduction

Numbers are omnipresent. In everyday life, we need to deal with money (e.g., comparing prices) or time (e.g., timetables for buses). Often number processing does not only include single-digit numbers but also multi-digit numbers. Thus, mastering multi-digit numbers is essential for everyday functioning. In the modern aging society, adults need these skills to autonomously manage their everyday lives up to old age. Unfortunately, most of the research on multi-digit number processing has been conducted with young adults, which cannot be generalized to older adults, because healthy aging might affect numerical skills (for a review see^1^). Interestingly, number processing is differentially affected by aging, as some functions in older adults show deficits (e.g., non-symbolic magnitude processing)^2^, and others might be preserved or even better developed (e.g., single-digit arithmetic;^3^) as compared to younger adults. As aging research is rather scarce in this field, this study aims to understand how multi-digit number processing changes during aging.

Fundamental representations of multi-digit numbers are commonly assumed to be magnitude and place-value (for a review see^4^). Representations of numerical magnitude are mostly assessed by the distance effect: Number comparison is faster when the distance between the numbers is larger (e.g., 6_1) than when it is smaller (e.g., 6_5)^5^. The distance effect was shown for single-digit^5^, two-digit^6^, three-digit^7^, and other multi-digit number comparison^8^. Thus, numerical distance effects occur in multiple number ranges, suggesting that magnitude representations are, in principle, present.

Multi-digit numbers are processed differently than single-digit numbers, as multi-digit numbers are not only processed in a holistic way but also decomposed according to the structure of the place-value system^4,9–11^. Thus, a multi-digit number is decomposed into its constituting digits with certain magnitudes (power values) ascribed to each digit depending on its position (place) within the number (e.g., 638 = 6 [hundreds] + 3 [decades] + 8 [units] = 6 × 100 + 3 × 10 + 8 × 1). This place-value activation^4^ is indicated, for instance, by the unit-decade compatibility effect in two-digit number comparison. The compatibility effect depicts that comparing two numbers takes longer when the (irrelevant) comparison of the units leads to a different decision relative to the comparison of the (decisive) decades (incompatible; e.g., 63_18 with 6 > 1 but 3 < 8) than when the comparison of both units and decades lead to the same decision (compatible; e.g., 68_23 with 6 > 2 and 8 > 3)^11^. The compatibility effect is even more pronounced when the units become more relevant (for instance by increasing the proportion of within-decade filler items such as 63_68)^12,13^.

Compatibility effects were found for the comparison of two-digit numbers^11,14^ as well as three-digit numbers^7,15,16^. Compatibility effects mostly reflect parallel processing of the unit and decade digits constituting multi-digit numbers^9,17^. For multi-digit number comparison beyond two- and three-digit numbers, the numbers seem to be decomposed into different chunks with parallel processing within chunks but sequential processing across chunks^18,19^. This holds especially for six-digit numbers for which compatibility effects were observed particularly at the beginning of the number^18^. In contrast, processing of four-digit numbers might be more influenced by interindividual differences, with some individuals showing a regular compatibility effect reflecting parallel processing and others showing a reverse compatibility effect rather reflecting sequential processing^18^ (for a mathematical elaboration of why a reverse compatibility effect indicates sequential processing see^20^). Taken together, the compatibility effect indicates place-value activation in two-digit number comparison and might be influenced by interindividual differences in four-digit number comparison.

Interindividual differences in number comparison were investigated in a large-scale web-based experiment^14^. Interestingly, age was found to be an important predictor for number processing. The skill to compare numbers seems to be preserved during healthy aging^21–23^ but not necessarily when cognitive deficits occur^24,25^. In general, healthy older adults are slower but more accurate in single-digit^21^ and two-digit number comparison^14,22^, indicating a speed-accuracy tradeoff between but not within the age groups^22^. The distance effect was shown to be larger for older than younger adults^21,22^; however, this only holds for reaction time, while the age influence on the distance effect in accuracy is rather smaller in older than younger adults^14,21, 22^. This may suggest that the number magnitude representation becomes more precise during aging because of lifetime experience with numbers, while processing speed in general decreases^26^. Here, we would not only like to replicate the speed-accuracy trade-off for number processing and the changes of the distance effect during aging, but also investigate whether such findings generalize to multiple number ranges. The reason is that it cannot be taken for granted that results from single-digit or even two-digit number processing generalize to multi-digit number processing as such^18^ (for reviews see^4,27^).

Concerning age effects in multi-digit number processing, the compatibility effect, which has become the hallmark effect indexing place-value activation, was only investigated in two-digit number comparison in a single study^14^. Crucially, the compatibility effect was not negatively influenced by aging^14^. Instead, older adults kept their high accuracy while younger adults made more mistakes when comparing incompatible number pairs under larger inhibition demands. However, the age distribution in this study was skewed with an overrepresentation of young adults and the compatibility effect was only examined in the two-digit number range.

The finding that older adults were better in comparing incompatible number pairs stands in marked contrast to the age-related deficit in inhibition^28^. When characterizing the compatibility effect as an interference effect (the irrelevant unit needs to be suppressed when comparing incompatible number pairs), an inhibition deficit would typically lead to larger interference effects—as shown for other effects in numerical cognition. For instance, the size-congruency effect was found to increase with age in a numerical Stroop task^29,30^. A possible explanation might be that aging only partly affects some inhibitory functions (such as executive inhibitory control required in Stroop tasks) but not others (such as automatic inhibitory control required in negative priming)^31,32^. Place-value activation in general was shown to be automatic^33^. In particular, the compatibility effect also has been shown to be rather automatic because it interacts with the size congruity effect^34^. As automaticity seems to increase with age in numerical cognition (automatized domain-specific number processing)^14^, one could argue that place-value integration and two-digit number processing in particular get more automatic with age and experience. Thus, the research question is two-sided, i.e., whether the compatibility effect increases with age due to deficits in inhibition (domain-general cognitive decline) or whether it decreases with age due to more automatized place-value integration.

This project addresses the question how aging influences number processing. In two studies, this question was investigated in adults of different age by focusing on the distance and compatibility effects in single-digit, two-digit, and four-digit number comparison.

### Study 1

This web-based experiment focuses on age-related effects in number processing across different number ranges. As preregistered [https://aspredicted.org/pu6nm.pdf], we state the following hypotheses regarding the replication of the numerical effects, the influence of age, and the measures of interest reaction time (RT) and accuracy (ACC):

- H1: The distance effect is expected for number comparison in general and the compatibility effect for multi-digit number comparison in particular.
- H2: Age-related effects are expected as increases in RT (due to typical age-related decreases in processing speed) and ACC in number comparison (generalization to multiple number ranges). The distance effect is expected to be smaller in ACC with increasing age and to be larger in RT with increasing age (generalization of two-digit numerical effects). With increasing age, the compatibility effect might be either larger (due to age-related deficits in inhibitory control) or smaller (due to more automatic place-value integration).

### Sample

The final German sample consisted of *N* = 195 adults (74 males, 121 females; age: *M* = 31.15, *SD* = 16.84, *Range* = 18–89 years). As preregistered [https://aspredicted.org/pu6nm.pdf], subjects were excluded from the initial sample of *N* = 424 who clicked the link due to the following criteria^35,36^: non-completion of the study (*n* = 185; including only *n* = 19 who started but not finished with a similar age distribution), age below 18 years (*n* = 0), not native in German (*n* = 18), study completion on tablet or smartphone (*n* = 3), very or extremely noisy environment during study completion (*n* = 2), responding dishonestly (*n* = 12). Out of the final sample, subjects with an overall accuracy below 66.7% were excluded for each number range (1-digit: *n* = 3, 2-digit: *n* = 2, 4-digit: *n* = 3), which reflects a rather liberal criterion when the chance level is 50% (i.e., excluding participants who responded correctly to about 1/3 and were at chance level for the other 2/3 [1/3 guessed correctly; 1/3 guessed incorrectly]). All subjects provided informed consent via mouse click. The study was approved by the Ethics Committee for Psychological Research of the University of Tuebingen (Germany) and conducted in accordance with the latest version of the Declaration of Helsinki.

### Number comparison

The symbolic number magnitude comparison task was conducted for three different number ranges: 1-digit, 2-digit, and 4-digit [open material: osf.io/3yqd4]. The factors distance (small, large) and compatibility (compatible, incompatible; only for multi-digit numbers) were orthogonally manipulated. Multi-digit number comparisons further included 33% filler items that differed only in one digit.

In the 1-digit number comparison task, the number pairs consisted of single-digit numbers (*Range*: 1–9) with small (1–4) or large distances (5–8). The stimuli included the same 16 number pairs, once with the larger number first (e.g., 8_3) and once reversed with the smaller number first (e.g., 3_8), with all of them presented twice. This resulted in 64 single-digit number pairs presented within one block.

In the 2-digit number comparison task, the number pairs consisted of two-digit numbers (*Range*: 21–98) with small (1–3) or large distances (4–7) between the decades. The stimuli were taken from Nuerk et al.^11^ and only included number pairs with large unit distances to get a larger compatibility effect. The stimuli included 120 unique between-decade items that were either compatible (e.g., 98_32 with 9 > 3 and 8 > 2) or incompatible (e.g., 92_38 with 9 > 3 but 2 < 8). Additionally, 60 within-decade filler items were added that only differed in the unit while the decade was the same (e.g., 92_96 with 9 = 9). The resulting 180 stimuli were separated into three blocks of 60 trials with each block containing 2/3 between-decade trials and 1/3 within-decade filler items to keep a proportion of 33% filler items in each block.

In the 4-digit number comparison task, the number pairs consisted of four-digit numbers (*Range*: 1423 bis 9847) that were constructed based on Meyerhoff et al.^18^. The numbers in a pair only differed in two successive digits, equally occurring in the beginning (e.g., **87**95_**32**95), in the middle (e.g., 5**79**8_5**24**8), or in the end (e.g., 14**87**_14**23**). These critical two-digit number pairs were taken from the 2-digit number comparison task and thus have the same properties, especially regarding distance and compatibility. As in 2-digit number comparison, the 120 stimuli were supplemented by 60 filler items that only differed in one digit and separated into three blocks of 60 trials with a proportion of 33% filler items.

Each critical number pair included two different numbers, distinct digits, no digit 0, and not the same digit twice (ties). Numerical size of each number, problem size, and the position of the larger number was counterbalanced. The numbers (58 px, Arial) were presented above each other (distance of 110 px) in white against a grey background; the numbers were horizontally jittered by one digit in the 2-digit number pairs and by one, two or three digits in the 4-digit number pairs. Each trial started with a fixation cross (20 px × 20 px) for 300 ms and the number pairs were presented until button press or a time limit of 5000 s. The subjects were asked to judge which of the numbers is larger; the instruction emphasized speed and accuracy. Subjects responded by pressing up or down arrow keys by the right or left index finger, respectively. The order of the seven blocks (1 × 1-digit, 3 × 2-digit, 3 × 4-digit) and the order of trials within each block was randomized. At the beginning, five practice trials were conducted including feedback. Reaction time (RT) and accuracy (ACC) serve as dependent measures.

### Procedure

The web-based experiment was programmed with PsychoPy^37^ and conducted on Pavlovia (pavlovia.org). The number comparison tasks were administered before the assessment of math anxiety. At the end, quality items were used to assess the amount of noise during study completion, honesty in responding to the study, and the used device [open material: osf.io/3yqd4] to ensure the data quality, as web-based experiments are conducted in less controllable environments^38^. The study duration was about 20 min in total.

### Analysis

Data analysis [open data and analysis: osf.io/3yqd4] was preregistered [https://aspredicted.org/pu6nm.pdf] and conducted using R (Version 4.2.3)^39^, RStudio (Version 1.2, RStudio, Inc., 2009–2020) and JASP (Jeffreys’s Amazing Statistics Program, Version 0.15, JASP Team, 2016). The analysis was conducted separately for 1-digit, 2-digit, and 4-digit number comparison tasks and separately for the dependent measures RT and ACC. Filler items were excluded from all analyses. For RT analysis, trials were further excluded if incorrectly solved (errors and missings; 1-digit: 3.38%, 2-digit: 4.32%, 4-digit: 6.33%), if RT below 200 ms (anticipations; 1-digit: 0.00%, 2-digit: 0.11%, 4-digit: 0.07%), if RT above 5000 ms (time out; 0%), or if RT deviating more than 3 median absolute deviations from the subject’s median in each task (1-digit: 5.08%, 2-digit: 4.26%, 4-digit: 2.20%). For exploratory analyses, RTs were additionally z-transformed to avoid interpreting confounds between effects and intraindividual variabilities, which can be a problem in developmental studies^40^.

For statistical data analysis, this study employs Bayesian hypothesis testing which allows to evaluate whether the observed data is more likely under the alternative hypothesis as compared to the null hypothesis (Bayes factor *BF*_*10*_ > 1) or vice versa compared (*BF*_*01*_ > 1, with *BF*_*01*_ = 1/*BF*_*10*_)^41^. Thereby, a *BF* between 1 and 3 indicates anecdotal evidence, between 3 and 10 moderate evidence, between 10 and 30 strong evidence, above 30 very strong evidence and above 100 extreme evidence in favor of one hypothesis^42^.

As confirmatory analyses, Bayesian ANCOVAs were conducted for each number range with the within-subject factors distance (small, large) and compatibility (compatible, incompatible; only for multi-digit numbers because single-digit numbers have no different place-values), the between-subject covariate age, and the interactions. Due to difficulties during data collection, math anxiety was not considered in the final analysis as originally planned (see preregistration at https://aspredicted.org/pu6nm.pdf; see Supplementary Material). The Bayesian ANCOVAs used a default Cauchy prior of *r* = 0.5, compared each model to the null model, and the effects were calculated by Bayesian model averaging considering matched models only (as suggested by Sebastiaan Mathôt). Post-hoc tests included Bayesian correlations with a stretched beta prior width of 1.0 and Bayesian paired *t*-tests with a Cauchy prior scale of 0.707. The distance effect was calculated as the difference between small and large distances and the compatibility effect as the difference between incompatible and compatible trials for RT and vice versa for ACC.

### Results

**Figure 1:**
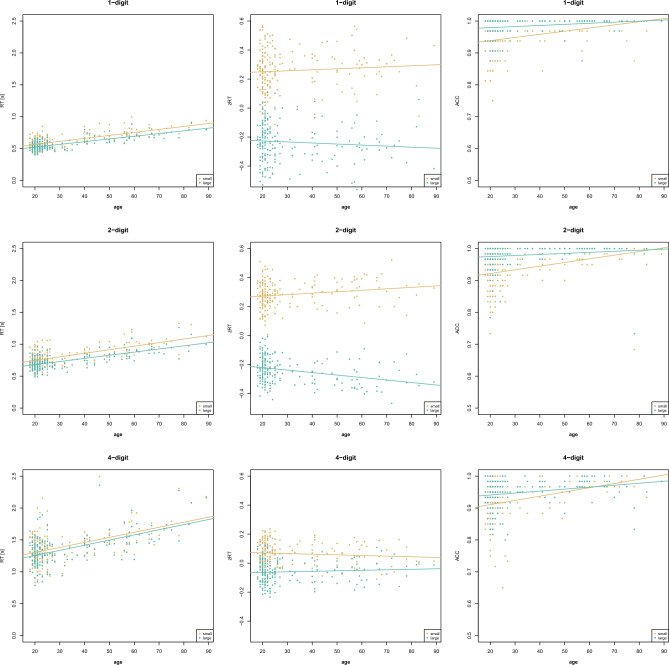
Age-related changes in the distance effect (Study 1).

**Figure 2:**
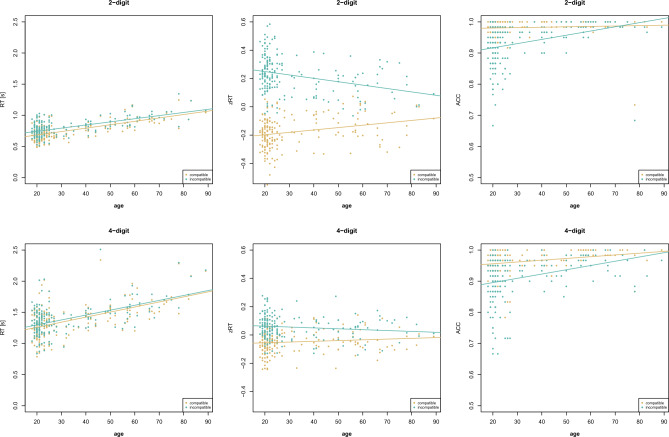
Age-related changes in the compatibility effect (Study 1).

Overall, evidence for main effects of distance, compatibility, and age was found for all number ranges (1-digit, 2-digit, 4-digit) and measures (RT, zRT, ACC), except for the impossible age effect in zRT (standardization with *M* = 0 and *SD* = 1 enables comparing standardized effect sizes, but overall means between groups are identical by definition). The distance effect indicates that number comparison is faster and more accurate for large than small distances. The compatibility effect indicates that number comparison is faster and more accurate for compatible than incompatible number pairs. Age effects indicated that with increasing age, individuals were slower but more accurate in number comparison. Moreover, different effects for interactions were found (for the results of the analysis of effects see Table 1, for visualizations see Figs. 1, 2) and post-hoc test results were reported in the text to supplement these findings [open analysis: osf.io/3yqd4].

**Table 1:** Results of Study 1.

Task	Measure	Effects	*P(incl)*	*P(incl|data)*	*BF*_*incl*_	*BF*_*excl*_
1-digit	RT	distance	0.400	0.503	> 100	
		age	0.400	0.503	> 100	
		distance × age	0.200	0.497	0.99	1.01
	zRT	distance	0.400	0.944	> 100	
		age	0.400	0.092	0.11	9.25
		distance × age	0.200	0.056	0.61	1.64
	ACC	distance	0.400	0.064	> 100	
		age	0.400	0.064	> 100	
		distance × age	0.200	0.936	14.58	
2-digit	RT	distance	0.263	< 0.001	> 100	
		compatibility	0.263	0.091	> 100	
		age	0.263	< 0.001	> 100	
		distance × compatibility	0.263	0.201	0.25	3.99
		distance × age	0.263	0.997	> 100	
		compatibility × age	0.263	0.735	2.78	
		distance × compatibility × age	0.053	< 0.001	< 0.01	> 100
	zRT	distance	0.263	< 0.001	> 100	
		compatibility	0.263	< 0.001	> 100	
		age	0.263	< 0.001	0.13	8.03
		distance × compatibility	0.263	0.731	17.18	
		distance × age	0.263	0.768	> 100	
		compatibility × age	0.263	0.773	> 100	
		distance × compatibility × age	0.053	0.227	0.31	3.20
	ACC	distance	0.263	< 0.001	> 100	
		compatibility	0.263	< 0.001	> 100	
		age	0.263	< 0.001	> 100	
		distance × compatibility	0.263	0.048	> 100	
		distance × age	0.263	0.048	> 100	
		compatibility × age	0.263	0.048	> 100	
		distance × compatibility × age	0.053	0.952	19.91	
4-digit	RT	distance	0.263	< 0.001	> 100	
		compatibility	0.263	< 0.001	> 100	
		age	0.263	0.957	> 100	
		distance × compatibility	0.263	1.000	> 100	
		distance × age	0.263	0.001	< 0.01	> 100
		compatibility × age	0.263	0.042	0.04	22.80
		distance × compatibility × age	0.053	< 0.001	< 0.01	> 100
	zRT	distance	0.263	< 0.001	> 100	
		compatibility	0.263	< 0.001	> 100	
		age	0.263	0.073	0.09	11.06
		distance × compatibility	0.263	0.997	> 100	
		distance × age	0.263	0.042	0.29	3.42
		compatibility × age	0.263	0.091	0.97	1.03
		distance × compatibility × age	0.053	0.003	0.16	6.10
	ACC	distance	0.263	0.039	> 100	
		compatibility	0.263	0.010	> 100	
		age	0.263	0.001	> 100	
		distance × compatibility	0.263	0.283	0.42	2.40
		distance × age	0.263	0.905	16.37	
		compatibility × age	0.263	0.946	65.84	
		distance × compatibility × age	0.053	0.039	0.15	6.68

Bayesian model averaging compared models with the effect with equivalent models without the effect, i.e., *P(excl)* = *P(incl)* and *P(excl|data)* = 1 – *P(incl|data)*. For interpretation, *BF*_*excl*_ was given when *BF*_*incl*_ < 1 with *BF*_*excl*_ = 1/*BF*_*incl*_*.*

In 1-digit number comparison, the best model for RT included the main effects of distance and age, *P(M)* = 0.200, *P(M|data)* = 0.503, *BF*_*M*_ = 4.04, *BF*_*10*_ > 100, error = 1.98%. The analysis of effects (RT) provided extreme evidence for the main effects of distance and age. The evidence for the interaction of distance and age was inconclusive. For zRT, the best model included only the main effect of distance, *P(M)* = 0.200, *P(M|data)* = 0.852, *BF*_*M*_ = 23.01, *BF*_*10*_ > 100, error = 3.65%. The analysis of effects (zRT) provided extreme evidence for the main effect of distance and was inconclusive regarding the interaction of distance and age. The best model for ACC included the main effects of distance and age as well as their interaction, *P(M)* = 0.200, *P(M|data)* = 0.936, *BF*_*M*_ = 58.30, *BF*_*10*_ > 100, error = 2.20%. The analysis of effects (ACC) provided extreme evidence for the main effect of distance and age, and strong evidence for the interaction of distance and age, indicating that the distance effect decreases with age, *r* =  − 0.220, *BF*_*10*_ = 9.52.

In 2-digit number comparison, the best model for RT included the main effects for distance, compatibility, and age as well as the interaction of distance and age and of compatibility and age, *P(M)* = 0.053, *P(M|data)* = 0.708, *BF*_*M*_ = 43.68, *BF*_*10*_ > 100, error = 15.87%. The analysis of effects (RT) provided extreme evidence for all main effects and the interaction of distance and age, indicating that the distance effect increases with age, *r* = 0.353, *BF*_*10*_ > 100. The evidence was inconclusive regarding the interaction of compatibility and age. There was extreme evidence against the three-way interaction and moderate evidence against the interaction of distance and compatibility. For zRT, the best model included all main effects and interactions except for the three-way interaction, *P(M)* = 0.053, *P(M|data)* = 0.726, *BF*_*M*_ = 47.70, *BF*_*10*_ > 100, error = 8.47%. The analysis of effects (zRT) provided extreme evidence for the main effects of distance and compatibility, for the interaction of distance and age, indicating that the distance effect increases with age, *r* = 0.258, *BF*_*10*_ = 60.95, and for the interaction of compatibility and age, indicating that the compatibility effect decreases with age, *r* =  − 0.293, *BF*_*10*_ > 100. Strong evidence was provided for the interaction of distance and compatibility, indicating that the compatibility effect is larger for large than small distances, *BF*_*10*_ > 100. There was moderate evidence against the three-way interaction. The best model for ACC was the full model with all main effects and interactions, *P(M)* = 0.053, *P(M|data)* = 0.952, *BF*_*M*_ > 100, *BF*_*10*_ > 100, error = 3.52%. The analysis of effects (ACC) provided extreme evidence for all main effects and interactions, except for the three-way interaction. The interaction of distance with age indicated that the distance effect decreases with age, *r* =  − 0.324, *BF*_*10*_ > 100, and the interaction of compatibility with age indicated that the compatibility effect decreases with age, *r* =  − 0.394, *BF*_*10*_ > 100.

In 4-digit number comparison, the best model for RT included the main effects for distance, compatibility, and age as well as the interaction of distance and compatibility, *P(M)* = 0.053, *P(M|data)* = 0.957, *BF*_*M*_ > 100, *BF*_*10*_ > 100, error = 14.14%. The analysis of effects (RT) provided extreme evidence for all main effects and the interaction of distance and compatibility, indicating that the compatibility effect is larger for small than large distances, *BF*_*10*_ = 4.50. There was extreme evidence against the interaction of distance and age and the three-way interaction, and strong evidence against the interaction of compatibility and age. For zRT, the best model included the main effects for distance and compatibility and their interaction, *P(M)* = 0.053, *P(M|data)* = 0.811, *BF*_*M*_ = 77.27, *BF*_*10*_ > 100, error = 2.36%. The analysis of effects (zRT) provided extreme evidence for the main effects of distance and compatibility and their interaction, indicating compatibility effect is larger for small than large distances, *BF*_*10*_ = 7.05. Evidence was inconclusive regarding the interaction of compatibility and age. There was moderate evidence against the interaction of distance and age and the three-way interaction. The best model for ACC included the main effects for distance, compatibility, and age as well as the interactions of distance and age and of compatibility and age, *P(M)* = 0.053, *P(M|data)* = 0.630, *BF*_*M*_ = 30.64, *BF*_*10*_ > 100, error = 2.43%. The analysis of effects (ACC) provided extreme evidence for all main effects. There was strong evidence for the interaction of distance with age, indicating that the distance effect decreases with age, *r* =  − 0.238, *BF*_*10*_ = 21.81, and very strong evidence for the interaction of compatibility and age, indicating that the compatibility effect decreases with age, *r* =  − 0.227, *BF*_*10*_ = 13.32. The evidence against the three-way interaction was moderate and against the interaction of distance and compatibility inconclusive.

In exploratory analysis, overall performance was found to increase from 1-digit over 2-digit to 4-digit number comparison.

### Discussion

Study 1 aimed at investigating age-related effects on the distance and compatibility effects in multiple number ranges. The results showed distance and compatibility effects in general, age effects on number comparison, and age-related changes in the distance and compatibility effects.

The distance and compatibility effects were found for all number ranges, thus replicating earlier studies and corroborating the validity of this online experiment. The distance effect indicates that speed as well as accuracy depends on the distance between the numbers that are compared^5–7^, which suggests that magnitude representations are relevant not only for single-digit and two-digit numbers but also for multi-digit numbers. The compatibility effect demonstrates that multi-digit numbers are processed in a decomposed way according to the place-value system^7,11, 14–16^, which could be generalized from two-digit numbers to multi-digit numbers, suggesting parallel processing of the constituting digits. While previous research on multi-digit number comparison found the compatibility effect mostly for six-digit numbers^18^, the current study observed it also for four-digit numbers. Taken together, distance and compatibility effects could be replicated, and magnitude and place-value representations could be generalized to all number ranges and age groups.

Concerning influences of age on general performance, speed in number comparison decreased while accuracy increased with increasing age in adults. This reflects the hypothesized speed-accuracy trade-off during aging: Older adults take more time to solve the task in order to solve it correctly, possibly due to risk aversion^21^. This leads to ceiling effects in accuracy for older adults and might be a reason why decreasing distance and compatibility effects were observed with increasing age in the accuracy analyses.

As expected, the distance effect increased with age concerning speed in two-digit number comparison but decreased with age concerning accuracy for all number ranges. The increasing distance effect could be only observed for two-digit number processing supporting previous findings^22^, but evidence was provided against a generalization to four-digit number processing. Moreover, evidence was too weak to indicate similar age-related effects for single-digit number processing that were previously found^21^. This is analogous to data in adult dyscalculics, where the distance effect differed from controls more in two-digit numbers than in single-digit numbers^43^. More generally, these data are consistent with the idea that such effects are more sensitive to group differences in multi-digit numbers, because they are less overlearned than single-digit numbers^4^. This suggests that two-digit number magnitude representations get more precise during adulthood because of increasing experience with numbers in the range up to 100.

The compatibility effect decreased with age for both two-digit and four-digit number comparison concerning accuracy. Concerning speed, evidence for an age-related decrease in the compatibility effect was obtained only for z-transformed reaction times in the two-digit number range^14^ and too weak to generalize to the four-digit number range. This shows that the effects are decreasing when controlling for the higher variance in speed of older adults. The age-related decrease (rather than increase) in the compatibility effect speaks for more automatic place-value integration due to age-related experience with multi-digit numbers rather than for an age-related deficit in inhibitory control.

Although the results are promising, a note of caution is due here regarding two aspects: First, the study was conducted as an online experiment and thus there might be a self-selection bias in the sample concerning older adults^44^. Second, the age distribution of the sample in Study 1 was limited by a lower number of older than younger adults so that the observed effects might reflect age-related changes in number processing during adulthood, but not necessarily aging effects at the end of lifespan development. These limitations were addressed in Study 2.

### Study 2

Study 2 aims at replicating the age-related changes in number processing found in Study 1 with a specific focus on aging. Due to the unequal age distribution, only *n* = 14 older adults above 66 years participated in Study 1. The follow-up study was conducted as a lab experiment with a sample of older adults which was compared to a sample of younger adults in the same number comparison task that was used in Study 1. As preregistered [https://aspredicted.org/bv9e7.pdf], we state the following hypotheses for this conceptual replication of Study 1:

- H1: The distance effect is expected for number comparison in general and the compatibility effect for multi-digit number comparison in particular.
- H2: Regarding age-related effects, older adults are expected to be generally slower (RT) in number comparison but more precise (ACC) in multi-digit number comparison as compared to younger adults. The distance effect is expected to be smaller in ACC (in number comparison in general) for older as compared to younger adults and to be larger in RT (only in multi-digit number comparison) for older as compared to younger adults. The compatibility effect is expected to be smaller in both ACC and RT in multi-digit number comparison for older as compared to younger adults.

### Sample

A Greek sample of *N* = 101 older adults (age above 66 years) and *N* = 102 younger adults (age between 18 and 36 years) was recruited for this study via printed announcements. The final sample consisted of *N* = 71 older adults (28 males, 43 females; age: *M* = 69.48, *SD* = 2.84, *Range* = 66–79 years) and *N* = 73 younger adults (23 males, 50 females; age: *M* = 22.69, *SD* = 3.56, *Range* = 18–36 years) after excluding subjects who did not complete the number comparison task [https://aspredicted.org/bv9e7.pdf]. All subjects were healthy, within the age range of the respective group, native in Greek, and conducted the experiment on a laptop or computer. The older adults had no cognitive impairment, as indicated by scores above the cut-off of 27 in the Mini Mental State Examination (MMSE)^45^: *M* = 29.10, *SD* = 0.72, *Range* = 28–30. Furthermore, the older adults showed no depression (cut-off of 7) according to the Geriatric Depression Scale (GDS-15)^46^: *M* = 1.86, *SD* = 1.60, *Range* = 0–6. Out of the final sample, subjects with an overall accuracy below 66.7% were excluded for each number range (1-digit: *n* = 0, 2-digit: *n* = 0, 4-digit: *n* = 14 older adults). All subjects provided written informed consent. The study was approved by the Research Ethics Committee of the University of Western Macedonia (Greece) and conducted in accordance with the latest version of the Declaration of Helsinki.

### Number comparison

The symbolic number magnitude comparison task was exactly the same as in Study 1 [open material: osf.io/3yqd4].

### Procedure

The study was conducted in a quiet room. First, the older adults were screened for cognitive impairments and depression. Then, all participants completed the number comparison task. Afterwards, participants were assessed in an arithmetic test (Math4Speed)^47^, an intelligence test and math anxiety questionnaire, that are not the focus of the current study (for results on math anxiety see Supplementary Material). The study duration was about 1 h in total.

### Analysis

Data analysis [open data and analysis: osf.io/3yqd4] was preregistered [https://aspredicted.org/bv9e7.pdf] and conducted using R^39^, RStudio (Version 1.2, RStudio, Inc., 2009–2020) and JASP (Jeffreys’s Amazing Statistics Program, Version 0.15, JASP Team, 2016). The analysis was conducted separately for 1-digit, 2-digit, and 4-digit number comparison tasks and separately for the dependent measures RT and ACC. Filler items were excluded from all analyses. For RT analysis, trials were further excluded if incorrectly solved (errors and missings, 1-digit: 1.51%, 2-digit: 6.83%, 4-digit: 8.18%), if RT below 200 ms (anticipations, 1-digit: 0.07%, 2-digit: 0.13%, 4-digit: 0.25%), if RT above 5000 ms (time out, 0%), or if RT deviating more than 3 median absolute deviations from the subject’s median in each task (1-digit: 12.06%, 2-digit: 7.15%, 4-digit: 1.45%). For exploratory analyses, RTs were additionally z-transformed.

As confirmatory analyses, Bayesian ANOVAs were conducted for each number range with the within-subject factors distance (small, large) and compatibility (compatible, incompatible; only for multi-digit numbers), the between-subject factor age (younger, older), and the interactions.

### Results

**Figure 3:**
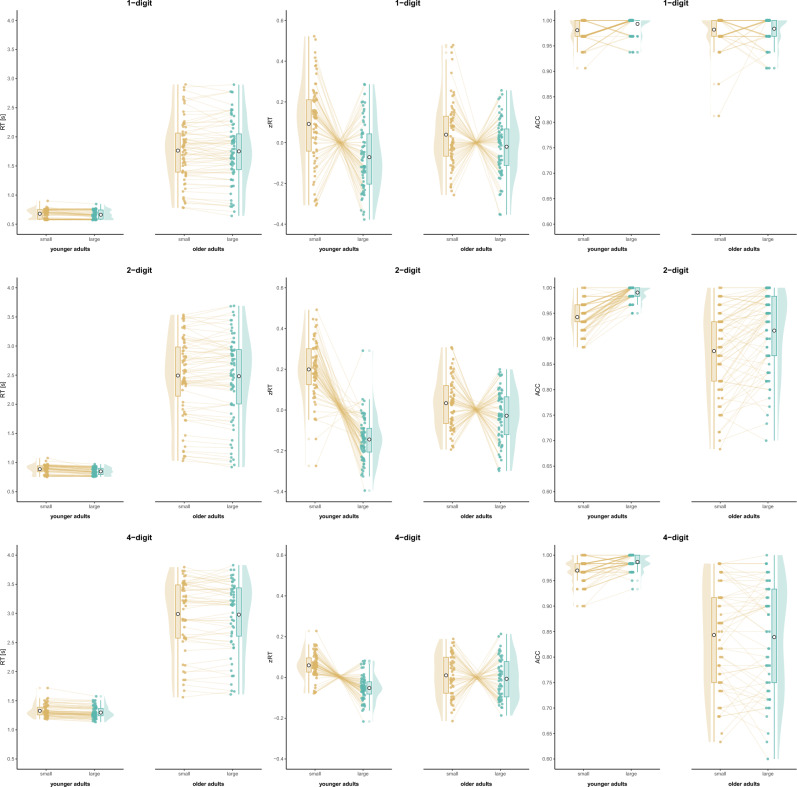
Age-related changes in the distance effect (Study 2).

**Figure 4:**
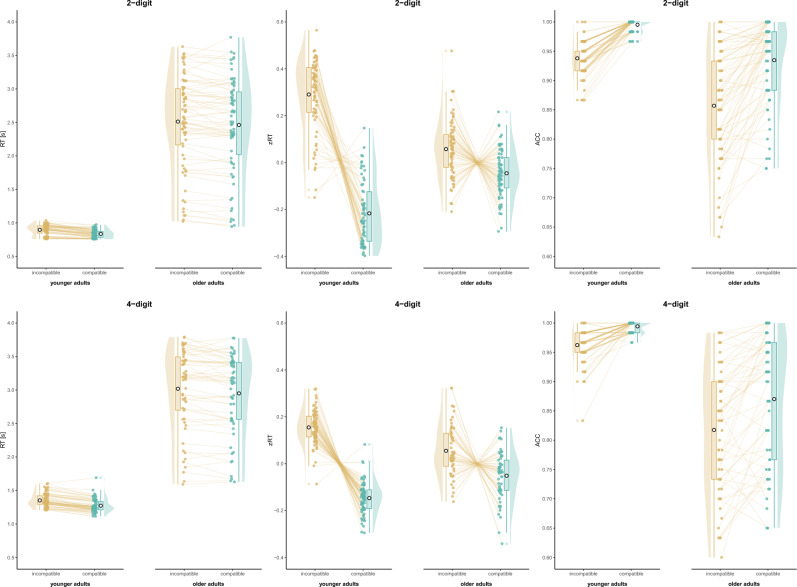
Age-related changes in the compatibility effect (Study 2).

Overall, evidence for distance and compatibility effects can be interpreted similar to Study 1; however, while evidence for age effects indicated that older adults were slower than younger adults (as in Study 1), older adults were also less accurate than younger adults in number comparison (contrary to Study 1). Thus, in Study 2, older adults performed worse in both measures and no speed-accuracy trade-off was observed. Moreover, different effects for interactions were found (for the results of the analysis of effects see Table 2, for visualizations see Figs. 3, 4) and post-hoc test results were reported in the text to supplement these findings [open analysis: osf.io/3yqd4].

**Table 2:** Results of Study 2.

Task	Measure	Effects	*P(incl)*	*P(incl|data)*	*BF*_*incl*_	*BF*_*excl*_
1-digit	RT	distance	0.400	0.340	0.57	1.75
		age	0.400	0.935	> 100	
		distance × age	0.200	0.065	0.19	5.26
	zRT	distance	0.400	0.698	> 100	
		age	0.400	0.085	0.14	7.23
		distance × age	0.200	0.302	3.56	
	ACC	distance	0.400	0.641	4.38	
		age	0.400	0.210	0.36	2.76
		distance × age	0.200	0.212	1.22	
2-digit	RT	distance	0.263	0.470	6.70	
		compatibility	0.263	0.633	> 100	
		age	0.263	0.618	> 100	
		distance × compatibility	0.263	0.256	0.38	2.61
		distance × age	0.263	0.271	0.42	2.41
		compatibility × age	0.263	0.141	0.17	6.06
		distance × compatibility × age	0.053	0.006	0.59	1.71
	zRT	distance	0.263	< 0.001	> 100	
		compatibility	0.263	< 0.001	> 100	
		age	0.263	< 0.001	0.25	4.08
		distance × compatibility	0.263	0.162	0.21	4.83
		distance × age	0.263	0.945	> 100	
		compatibility × age	0.263	0.945	> 100	
		distance × compatibility × age	0.053	0.055	0.34	2.92
	ACC	distance	0.263	< 0.001	> 100	
		compatibility	0.263	< 0.001	> 100	
		age	0.263	0.223	> 100	
		distance × compatibility	0.263	0.962	> 100	
		distance × age	0.263	0.163	0.20	4.90
		compatibility × age	0.263	0.694	2.58	
		distance × compatibility × age	0.053	0.038	0.32	3.13
4-digit	RT	distance	0.263	0.240	3.24	
		compatibility	0.263	0.325	> 100	
		age	0.263	0.680	> 100	
		distance × compatibility	0.263	0.587	1.97	
		distance × age	0.263	0.180	0.26	3.92
		compatibility × age	0.263	0.124	0.15	6.74
		distance × compatibility × age	0.053	0.041	2.43	
	zRT	distance	0.263	< 0.001	> 100	
		compatibility	0.263	< 0.001	> 100	
		age	0.263	< 0.001	0.12	8.45
		distance × compatibility	0.263	< 0.001	> 100	
		distance × age	0.263	< 0.001	33.29	
		compatibility × age	0.263	< 0.001	> 100	
		distance × compatibility × age	0.053	1.000	> 100	
	ACC	distance	0.263	0.066	0.42	2.39
		compatibility	0.263	0.214	> 100	
		age	0.263	0.308	> 100	
		distance × compatibility	0.263	0.648	3.90	
		distance × age	0.263	0.512	1.70	
		compatibility × age	0.263	0.313	0.48	2.10
		distance × compatibility × age	0.053	0.029	0.27	4.42

Bayesian model averaging compared models with the effect with equivalent models without the effect, i.e., *P(excl)* = *P(incl)* and *P(excl|data)* = 1 – *P(incl|data)*. For interpretation, *BF*_*excl*_ was given when *BF*_*incl*_ < 1 with *BF*_*excl*_ = 1/*BF*_*incl*_. The analysis for reaction time (RT) and accuracy (ACC) were confirmatory, the analysis for z-transformed RT (zRT) was exploratory.

In 1-digit number comparison, the best model for RT included only the main effect of age, *P(M)* = 0.200, *P(M|data)* = 0.595, *BF*_*M*_ = 5.88, *BF*_*10*_ > 100, error = 0.81%. The analysis of effects (RT) provided extreme evidence for age but moderate evidence against the interaction of age and distance. The evidence was inconclusive regarding the distance effect. For zRT, the best model included only the main effect of distance, *P(M)* = 0.200, *P(M|data)* = 0.613, *BF*_*M*_ = 6.34, *BF*_*10*_ > 100, error = 0.79%. The analysis of effects (zRT) provided extreme evidence for distance effect and moderate evidence for the interaction of distance and age, indicating that older adults showed a smaller distance effect than younger adults; however, the post-hoc test did not provide evidence for this effect, *BF*_*10*_ = 0.77. The best model for ACC included the main effect of distance, *P(M)* = 0.200, *P(M|data)* = 0.468, *BF*_*M*_ = 3.52, *BF*_*10*_ = 4.23, error = 1.30%. The analysis of effects (ACC) provided moderate evidence for the distance effect. The evidence was inconclusive regarding the main effect of age and the interaction.

In 2-digit number comparison, the best model for RT included the main effects for distance, compatibility, and age, *P(M)* = 0.053, *P(M|data)* = 0.401, *BF*_*M*_ = 12.05, *BF*_*10*_ > 100, error = 1.79%. The analysis of effects (RT) provided extreme evidence for compatibility and age, and moderate evidence for distance. The evidence was moderate against the interaction of compatibility and age, and inconclusive for all other interactions. For zRT, the best model included all main effects and the interactions of distance and age and of compatibility and age, *P(M)* = 0.053, *P(M|data)* = 0.783, *BF*_*M*_ = 64.79, *BF*_*10*_ > 100, error = 3.93%. The analysis of effects (zRT) provided extreme evidence for the main effects of distance and compatibility, the interaction of distance and age (indicating that older adults showed a smaller distance effect than younger adults, *BF*_*10*_ > 100), and the interaction of compatibility and age (indicating that older adults showed a smaller compatibility effect than younger adults, *BF*_*10*_ > 100). Evidence was moderate against the interaction of distance and compatibility, and inconclusive regarding the three-way interaction. The best model for ACC included main effects for distance, compatibility, and age as well as the interactions of distance and compatibility and of compatibility and age, *P(M)* = 0.053, *P(M|data)* = 0.576, *BF*_*M*_ = 24.46, *BF*_*10*_ > 100, error = 4.96%. The analysis of effects (ACC) provided extreme evidence for all main effects and the interaction of distance and compatibility, indicating that the compatibility effect is larger for large than small distances, *BF*_*10*_ > 100. The evidence was moderate against the interaction of distance and age and the three-way interaction, and inconclusive for the interaction of compatibility and age.

In 4-digit number comparison, the best model for RT included the main effects for distance, compatibility, and age as well as the interaction of distance and compatibility, *P(M)* = 0.053, *P(M|data)* = 0.405, *BF*_*M*_ = 12.27, *BF*_*10*_ > 100, error = 3.38%. The analysis of effects (RT) provided extreme evidence for compatibility and age, and moderate evidence for distance. There was moderate evidence against the interactions of distance and age and of compatibility and age. The evidence was inconclusive regarding the interaction of distance and compatibility and the three-way interaction. For zRT, the best model was the full model with all main effects and interactions, *P(M)* = 0.053, *P(M|data)* = 1.00, *BF*_*M*_ > 100, *BF*_*10*_ > 100, error = 13.14%. The analysis of effects (zRT) provided extreme evidence for the main effects of distance and compatibility, and their interaction, indicating that the compatibility effect is larger for large than small distances, *BF*_*10*_ > 100. Extreme evidence was also provided for the three-way interaction and the interaction of compatibility and age, indicating that older adults showed a smaller compatibility effect than younger adults, *BF*_*10*_ > 100. Very strong evidence was provided for the interaction of distance and age, indicating that older adults showed a smaller distance effect than younger adults, *BF*_*10*_ = 26.88. The best model for ACC included the main effects for distance, compatibility, and age as well as the interactions of distance and compatibility and of distance and age, *P(M)* = 0.053, *P(M|data)* = 0.283, *BF*_*M*_ = 7.10, *BF*_*10*_ > 100, error = 6.31%. The analysis of effects (ACC) provided extreme evidence for compatibility and age, and moderate evidence for the interaction of distance and compatibility, indicating that the compatibility effect is larger for large than small distances, *BF*_*10*_ = 4.39. The evidence against the three-way interaction was moderate and inconclusive for the distance effect as well as for the interactions of distance and age and of compatibility and age.

In exploratory analysis, overall performance was found to increase from 1-digit over 2-digit to 4-digit number comparison. Furthermore, interindividual influences of math skills on the distance and compatibility effects were explored. However, we found no evidence for correlations between the distance and compatibility effects with addition, subtraction, multiplication, or division skills as indicated by the Math4Speed, considering younger and older adults separately.

### Discussion

Study 2 aimed at replicating the age-related effects on the distance and compatibility effects in multiple number ranges with a specific focus on aging. The results showed that the distance and compatibility effects could mostly be replicated, while age effects are partly in line with and partly differ from Study 1.

In general, older adults were slower in number comparison than younger adults, as it was found in Study 1. However, older adults were not more but less accurate in multi-digit number comparison. This might be explained by the higher age of older adults that might be associated with a general decrease in performance instead of a speed-accuracy trade-off. As this study does not show ceiling effects in accuracy for older adults and could not replicate the age-related effects in the distance and compatibility effects for accuracy, the age-related decrease of these effects in Study 1 might also be explained by a lack of variance rather than a developmental change. A further reason for diverging results might be sampling: While probably rather cognitively fitter older adults participate in online studies as Study 1, the sample in Study 2 was recruited at senior centers.

Although the distance and compatibility effects could mostly be replicated, there was one exception: For single-digit numbers, the data was inconclusive concerning the distance effect. We explored whether this was due to the conservative outlier exclusion method (*Median* RT ± 3 *MAD*) which resulted in a relatively high exclusion rate of trials (> 10%) in this particular case. However, even when reanalyzing the data with a more liberal exclusion method (*Mean* RT ± 3 *SD*) which resulted in a low exclusion rate of trials (< 1%), the results stayed substantially the same. Despite the inconclusive distance effect for overall RT, zRT consistently revealed a distance effect across studies.

Regarding the effects of interest, age-related changes of the distance and compatibility effects were observed only for z-transformed reaction times, but consistently across all number ranges. Surprisingly, the distance effect decreased with age. This finding is contrary to the results of Study 1 and previous studies^21,22^, who did not use a standardized measure to detect age differences. Thus, number magnitude processing might be affected by aging in older age (Study 2), while it was rather preserved or even more pronounced until middle-aged adulthood (Study 1). As this aging effect was consistent across number ranges in Study 2, and as the design of Study 2 was superior to Study 1 in detecting aging effects, a generalization from single-digit to multi-digit number processing can be assumed.

Concerning place-value processing, the compatibility effect decreased with age for both two-digit and four-digit number comparison concerning accuracy as expected, thus replicating and strengthening the results of Study 1. Therefore, both studies consistently confirm a more automatic place-value integration due to age-related experience with multi-digit numbers even for older age. The inhibition hypothesis, which would have predicted a larger compatibility effect in older adults, can be refuted for place-value integration in multi-digit number processing.

We wish to note that the different results of both studies are not necessarily contradictory, as they also differ in other aspects – these are addressed in the following general discussion.

## General discussion

This research aimed at investigating the role of aging in number processing. The main consistent findings of the two studies were that number processing slows down and the compatibility effect decreases with increasing age, suggesting some more automatic place-value processing. Some inconsistent findings concern the different patterns for accuracy: Older adults either showed a speed-accuracy trade-off (Study 1) or general lower performance (Study 2) than younger adults. What is more, the conclusions about changes in magnitude representation differed between the two studies and depended on the measure.

During adulthood, the speed of number processing decreases, reflecting the general decrease in processing speed during aging^26^. Across both studies and all number ranges, we also found that older adults were consistently slower in number magnitude comparison than younger adults. Thus, the general decrease of processing speed also applies to multi-digit number processing.

Age-related changes in place-value processing were assessed by the compatibility effect. Two opposite hypotheses could be assumed in the general introduction: (1) an inhibition deficit hypothesis, which would lead to a larger compatibility effect in older adults, because the irrelevant and interfering unit digit cannot be inhibited as well as in younger adults; (2) an automatization hypothesis, which would suggest more automatic place-value integration with longer experience, leading to a smaller compatibility effect in older adults. The compatibility effect was consistently shown to decrease with age for both two-digit and four-digit numbers (Study 1 and 2), supporting the automatization hypothesis. While such age-related changes in accuracy might be attributed to ceiling effects (Study 1, see below), this does not explain the effects in standardized speed. Therefore, the compatibility effect is driven by more automatized place-value integration due to lifelong experience^14^ and not affected by the general cognitive decline leading to a deficit in inhibitory control.

The results of our studies revealed two important differences. First, the age-related speed-accuracy trade-off (Study 1)^21,22^ might be specific to the selection bias for sampling older adults in online studies^44^ as it did not replicate in a broader sample of older adults (Study 2). The age-related decline in performance also affected accuracy.

Second, age-related changes in number magnitude processing were assessed by the distance effect. The results, however, were not consistent across the studies. In Study 1, the expected speed-accuracy trade-off during aging revealed a decreasing distance effect in accuracy and an increasing distance effect in speed for two-digit numbers. In line with previous studies^21,22^, this finding can be explained by preserved number magnitude representations. In contrast, an age-related decrease in the distance effect was found when controlling for the higher variance in older adults by using standardization of speed (Study 2). This suggests that aging might indeed affect basic symbolic number skills, probably more so in older age, as it was shown for non-symbolic number skills^2,23^.

Finally, we address the differences between Study 1 and 2, as well as some previous studies, and discuss their implications for future studies.

1. The difference in the age distribution with older adults being slightly older in Study 2 than in Study 1 might explain why accuracy increased in Study 1 but decreased in Study 2. Both results might reflect the underlying age distribution, indicating that the decrease in accuracy might start relatively late during aging, especially for rather simple tasks such as number magnitude comparison. Furthermore, this led to the ceiling effects in accuracy for older adults which were observed in Study 1 but not Study 2 and consequently smaller distance and compatibility effects during aging. Hence, accuracy effects should be interpreted with care.
2. Differences in the study design and measures might lead to differences in the statistical analyses. The study design included age as a continuous between-subject factor (Study 1) or two extreme groups of age (Study 2). Continuous linear methods might be biased by the distribution, while group comparisons might obscure interindividual variances within groups. Regarding the analyzed measure, previous studies did not analyze zRT, but only overall RT. For overall RT, few participants with high intraindividual variability tend to drive effects^40^. This is a problem, because standard statistical procedures generally assume independent, identically distributed variables within each group with the same mean and variance within each group and condition. As this might be violated for older adults, effects for RT might be blurred in lifespan research. Therefore, z-transformation might help to detect or even invert age-related effects^40^.
3. The samples between the two studies differed in their cultural background (Germany vs. Greece) and their language (with vs. without number word inversion—in German two-digit number words are spoken inverted, e.g., “one-and-twenty” for 21). Regarding culture, education might play an important role in numerical skills possibly explaining cultural differences between the studies. However, the compatibility effect seems to be rather unrelated to education. On the one hand, the compatibility effect was shown even in semi-illiterates with almost no schooling^48^. This indicates that the effect does not critically depend on high or low math education. On the other hand, the compatibility effect could still be modulated by education. However, the compatibility effect does not correlate with education years^48^ and does not differ between groups with different educational backgrounds^49^. Although this does not theoretically exclude that education might play a role in number comparison, the literature so far has failed to find any impact of education on the compatibility effect. Regarding language, number word inversion has been consistently shown to affect the compatibility effect in various lab studies^49–51^ (for a review see^52^), but not in an online study^14^. Furthermore, interindividual variation in parallel vs. sequential processing is larger in languages with inversion^15^. In the current study, compatibility effects were consistently observed even in a language without inversion (Greek), however, direct cross-cultural comparisons are needed for further insights. Moreover, cultural and language effects were less commonly found in web-based studies, maybe due to the English context in the internet^14^.
4. Due to the different setting of the studies (online experiment vs. lab setting) the recruitment of the samples might have targeted older adults who are more vs. less experienced in using computers. For instance, older adults in Study 2 were less familiar with computer use, but at least cognitive screening ensured the focus on healthy aging. Whenever possible, cognitive screening should be done for all age groups measured in different studies to characterize cognitive performance levels in different samples.

In summary, basic symbolic numerical skills are generally preserved during healthy aging, but processing multi-digit numbers gets slower with age, in line with a general decline in processing speed. Place-value integration gets more automatized with increasing age and thus automatization seems more relevant than an eventual inhibition deficit for multi-digit numbers. The results on age-related changes in number magnitude representations differed between studies, depending on sampling, design, measures, and analyses used. We can conclude that the decrease of processing speed and the automatization of place-value integration is consistently supported by the data, while further research is needed to understand age-related changes of number magnitude representations (as indexed by the distance effect).

### Supplementary Information

Supplementary Information.

## Data Availability

The datasets generated and analyzed for the current study are available at OSF [osf.io/3yqd4].
