# Supplementary material for: Age-related effects in magnitude and place-value processing
Source: Sci Rep. 2024 Jun 13;14:13645. doi: 10.1038/s41598-024-63298-z (PMC11176331; doi:10.1038/s41598-024-63298-z)
Supplement: Supplementary file 1 — Supplementary Information. [file 41598_2024_63298_MOESM1_ESM.pdf]

Supplementary Material on Math Anxiety

The original idea of the study was to also investigate the influence of math anxiety – besides age – on number processing. However, due to technical problems for the math anxiety questionnaire in the online experiment, the math anxiety data was not valid and thus, the focus on math anxiety was removed from the study. In the follow-up experiment, math anxiety data was assessed but analyses were not preregistered. Here, we introduce the theoretical background and our hypotheses, describe the used questionnaire and the problems in data collection, and report the exploratory results on how math anxiety influences number processing.

Introduction

Besides age, interindividual differences in math anxiety also influence performance in numerical tasks (for overviews see Barroso et al., 2020; Dowker et al., 2016). However, the negative impact of math anxiety was mostly shown for math performance and less for basic number processing. Nevertheless, individuals with high math anxiety were found to be slower in comparing single-digit numbers to reach similar accuracy levels as individuals with low math anxiety (Maloney et al., 2011; Núñez-Peña & Suárez-Pellicioni, 2014; but see Dietrich et al., 2015). Moreover, the distance effect was observed to be larger with higher levels of math anxiety (for reaction time but not accuracy; Dietrich et al., 2015; Maloney et al., 2011; Núñez-Peña & Suárez-Pellicioni, 2014). While this finding was originally interpreted as a less precise number magnitude representation in individuals with high math anxiety (Maloney et al., 2011; Núñez-Peña & Suárez-Pellicioni, 2014), a study by Dietrich et al. (2015) found no impact of math anxiety on the distance effect in non-symbolic number comparison and thus attributed the role of math anxiety in symbolic number comparison to the impairment of comparison processes. If even single-digit number comparison is affected by math anxiety, what about multi-digit number comparison? According to the anxiety-complexity effect (Ashcraft & Faust, 1994; Huber & Artemenko, 2021), the impact of math anxiety might even increase with the complexity of the numerical task – but it was not yet investigated for multi-digit number comparison.

Similarly, the role of math anxiety in the compatibility effect in multi-digit number comparison is not clear. As the compatibility effect can be characterized as a unit inference effect, inhibition of irrelevant units is necessary when comparing incompatible number pairs, especially in the context of within-decade fillers. Like age, math anxiety is related to deficits in inhibition (e.g., Hopko et al., 1998), and thus distraction by task-irrelevant stimuli impairs performance, as assumed by the attention control theory (Eysenck et al., 2007). Therefore, math anxiety might be associated with larger compatibility effects, which will be tested in the current study. There is only one study investigating the impact of math anxiety on the compatibility effect: Although Pletzer et al. (2015) found no alternations of the compatibility effect due to math anxiety on a behavioral level, the neural processing efficiency especially in inhibitory brain areas was impaired by math anxiety, leading to no neural compatibility effect in individuals with high math anxiety. Thus, the inhibition deficit related to math anxiety might lead to larger compatibility effects on a behavioral level when tested in larger sample.

Hypothesis

In the preregistration of Study 1 [https://aspredicted.org/pu6nm.pdf], we stated the following hypothesis regarding the influence of math anxiety on number processing: Math anxiety-related effects are expected as increases in RT and decreases in ACC in multi-digit number comparison. (Note that this deviates from the preregistration because it needed to be corrected. In the preregistration, “math anxiety-related effects are

expected as decreases in RT and ACC" was a typo, as we also stated that "increasing math anxiety is associated with lower numerical task performance" which actually means increased RT and decreased ACC.) The distance effect is expected to be larger in RT with increasing math anxiety (generalization of single-digit numerical effects). The compatibility effect is expected to be larger with increasing math anxiety (due to anxiety-related deficits in inhibitory control).

Methods

Math anxiety was assessed by the abbreviated math anxiety scale (AMAS; Hopko et al., 2003) for Study 1 in its German translation (Artemenko et al., 2021) and for Study 2 in its Greek translation (Klados et al., 2017). Subjects were asked to indicate for 9 items on a 5-point Likert scale (1 = low anxiety, 5 = high anxiety) how anxious they would feel in the math situations described. The AMAS was previously shown to be appropriate for web-based research on math anxiety (Cipora et al., 2018). The reliability of the AMAS in the Greek sample was Cronbach's $\alpha = 0.98$ for younger adults and $\alpha = 0.98$ for older adults, respectively. Math anxiety was assessed after the number comparison tasks. Mean values were calculated with higher values indicating higher levels of math anxiety.

Analysis

In Study 1, Bayesian ANCOVAs were planned as confirmatory analyses for each number range with the within-subject factors distance (small, large) and compatibility (compatible, incompatible; only for multi-digit numbers), the between-subject covariates age and math anxiety, and the interactions. Due to difficulties during data collection, math anxiety was excluded from the analysis and therefore the reported analysis in the paper deviates from the preregistration [https://aspredicted.org/pu6nm.pdf]. The reason was that participants reported that they were not always able to select the options on the Likert scale but that the middle point of the scale was automatically logged. Consequently, the distribution of the responses in the questionnaire showed an overrepresentation of the middle option and thus should not be analyzed.

In Study 2, analyses on math anxiety were not preregistered so that exploratory analyses were conducted to explore the influence of math anxiety on number processing.

Results

In Study 2, younger and older adults were compared in math anxiety by a Bayesian independent sample *t*-test first. Results indicate that older adults ($M = 2.97$; $SD = 1.09$) reported significantly lower levels of math anxiety than younger adults ($M = 3.98$; $SD = 0.94$), $BF_{10} > 100$. Therefore, the following Bayesian ANCOVAs on the influence of math anxiety on number processing were conducted only for younger adults (for the results of the analysis of effects see Table S1, for Open Analysis and Open Data see osf.io/3yqd4).

For 1-digit number comparison, the best model for RT included the main effects for distance and math anxiety, $P(M) = 0.200$, $P(M|data) = 0.743$, $BF_M = 11.56$, $BF_{10} > 100$, error = 3.50%. The analysis of effects provided extreme evidence for the distance effect, moderate evidence for the effect of math anxiety, and moderate evidence against the interaction.

For 2-digit number comparison, the best model for RT included the main effects for distance, compatibility, and math anxiety, $P(M) = 0.053$, $P(M|data) = 0.329$, $BF_M = 8.83$, $BF_{10} > 100$, error = 11.48%. The analysis of effects provided extreme evidence for the distance effect and the compatibility effect. The evidence for the effect of math anxiety and against the interaction of compatibility and math anxiety were inconclusive. There was moderate evidence against the interaction of distance and math anxiety, the interaction of distance and compatibility, and the three-way interaction.

For 4-digit number comparison, the best model for RT included the main effects for distance and compatibility and their interaction, *P(M)* = 0.053, *P(M|data)* = 0.586, BF_M = 25.46, BF_{10} > 100, error = 2.47%. The analysis of effects provided extreme evidence for the distance effect, the compatibility effect, and their interaction. The evidence against all other effects was inconclusive.

The results for zRT and ACC were similar to RT (for Open Analysis see osf.io/3yqd4). In summary, the influence of math anxiety on the distance and compatibility effects was not found in younger adults.

Table S1. Results for math anxiety for younger adults in Study 2.

Task	Measure	Effects	*P(incl)*	*P(incl|data)*	BF_{incl}	BF_{excl}
1-digit	RT	distance	0.400	0.919	> 100	
		math anxiety	0.400	0.743	4.21	
		distance × math anxiety	0.200	0.080	0.11	9.26
2-digit	RT	distance	0.263	0.704	> 100	
		compatibility	0.263	0.654	> 100	
		math anxiety	0.263	0.408	1.28	
		distance × compatibility	0.263	0.209	0.26	3.79
		distance × math anxiety	0.263	0.112	0.20	5.08
		compatibility × math anxiety	0.263	0.195	0.40	2.48
		distance × compatibility × math anxiety	0.053	0.002	0.31	3.20
4-digit	RT	distance	0.263	< 0.001	> 100	
		compatibility	0.263	< 0.001	> 100	
		math anxiety	0.263	0.219	0.37	2.68
		distance × compatibility	0.263	0.991	> 100	
		distance × math anxiety	0.263	0.104	0.34	2.91
		compatibility × math anxiety	0.263	0.103	0.34	2.95
		distance × compatibility × math anxiety	0.053	0.009	0.46	2.16

Note. Bayesian model averaging compared models with the effect with equivalent models without the effect, i.e., *P(excl)* = *P(incl)* and *P(excl|data)* = 1 – *P(incl|data)*. For interpretation, BF_{excl} was given when BF_{incl} < 1 with BF_{excl} = 1/BF_{incl}.

References

Artemenko, C., Masson, N., Georges, C., Nuerk, H.-C., & Cipora, K. (2021). Not all elementary school teachers are scared of math. *Journal of Numerical Cognition*.

Ashcraft, M. H., & Faust, M. W. (1994). Mathematics anxiety and mental arithmetic performance: An exploratory investigation. *Cognition & Emotion, 8*(2), 97–125. https://doi.org/10.1080/02699939408408931

Barroso, C., Ganlex, C. M., McGraw, A. L., Geer, E. A., Hart, S. A., & Daucourt, M. C. (2020). A Meta-analysis of the Relation Between Math Anxiety and Math Achievement Connie. *Psychological Bulletin*. https://doi.org/10.1037/bul0000307

Cipora, K., Willmes, K., Szwarc, A., & Nuerk, H.-C. (2018). Norms and validation of the online and paper-and-pencil versions of the Abbreviated Math Anxiety Scale (AMAS) for Polish adolescents and adults. *Journal of Numerical Cognition*, *3*(3), 667–693. https://doi.org/10.5964/jnc.v3i3.121

Dietrich, J. F., Huber, S., Moeller, K., & Klein, E. (2015). The influence of math anxiety on symbolic and non-symbolic magnitude processing. *Frontiers in Psychology*, *6*(OCT). https://doi.org/10.3389/fpsyg.2015.01621

Dowker, A., Sarkar, A., & Looi, C. Y. (2016). Mathematics anxiety: What have we learned in 60 years? *Frontiers in Psychology*, *7*. https://doi.org/10.3389/fpsyg.2016.00508

Eysenck, M. W., Derakshan, N., Santos, R., & Calvo, M. G. (2007). Anxiety and cognitive performance: attentional control theory. *Emotion (Washington, D.C.)*, *7*(2), 336–353. https://doi.org/10.1037/1528-3542.7.2.336

Hopko, D., Ashcraft, M., & Gute, J. (1998). Mathematics anxiety and working memory: Support for the existence of a deficient inhibition mechanism. *Journal of Anxiety Disorders*, *12*(4), 343–355.

Hopko, D., Mahadevan, R., Bare, R., & Hunt, M. (2003). The Abbreviated Math Anxiety Scale (AMAS): Construction, Validity, and Reliability. *Assessment*, *10*(2), 178–182. https://doi.org/10.1177/1073191103010002008

Huber, J. F., & Artemenko, C. (2021). Anxiety-related difficulties with complex arithmetic – A web-based replication of the anxiety-complexity effect. *Zeitschrift Für Psychologie*.

Klados, M. A., Pandria, N., Micheloyannis, S., Margulies, D., & Bamidis, P. D. (2017). Math anxiety: Brain cortical network changes in anticipation of doing mathematics. *International Journal of Psychophysiology*, *122*, 24–31. https://doi.org/10.1016/j.ijpsycho.2017.05.003

Maloney, E. A., Ansari, D., & Fugelsang, J. A. (2011). The effect of mathematics anxiety on the processing of numerical magnitude. *Quarterly Journal of Experimental Psychology (2006)*, *64*(1), 10–16. https://doi.org/10.1080/17470218.2010.533278

Núñez-Peña, M. I., & Suárez-Pellicioni, M. (2014). Less precise representation of numerical magnitude in high math-anxious individuals: an ERP study of the size and distance effects. *Biological Psychology*, *103*, 176–183. https://doi.org/10.1016/j.biopsycho.2014.09.004

Pletzer, B., Kronbichler, M., Nuerk, H.-C., & Kerschbaum, H. H. (2015). Mathematics anxiety reduces default mode network deactivation in response to numerical tasks. *Frontiers in Human Neuroscience*, *9*(202). https://doi.org/10.3389/fnhum.2015.00202
